# Revolutionizing cancer treatment with *Halomonas Aquamarina* L-Glutaminase: insights from in vitro and computational studies

**DOI:** 10.1038/s41598-025-14230-6

**Published:** 2025-08-24

**Authors:** Sara Abdelsayed, Alaa Elmetwalli, Jihan Hassan, Mohamed O. Abdel Monem, Ali H. El-Far, Fuad Ameen, Mervat G. Hassan

**Affiliations:** 1 Botany and Microbiology Department, Faculty of Science, Benha University, Benha, Egypt; 2 Department of Clinical Trial Research Unit and Drug Discovery, Egyptian Liver Research Institute and Hospital (ELRIAH), Mansoura, Egypt; 3 Prince Fahad bin Sultan Chair for Biomedical Research, University of Tabuk, Tabuk, Saudi Arabia; 4 Department of Applied Medical Chemistry, Medical Research Institute, Alexandria University, Alexandria, Egypt; 5 Key Laboratory of Epigenetics and Oncology, The Research Center for Preclinical Medicine, Southwest Medical University, Luzhou, 646000 China; 6 Department of Botany and Microbiology, College of Science, King Saud University, Riyadh 11451, P.O. Box 2455, Saudi Arabia

**Keywords:** Enzyme activity, *Halomonas Aquamarina*, L-glutaminase, Liver cancer, Tannic acid, Biological techniques, Biomaterials, Environmental biotechnology

## Abstract

Bacterial L-glutaminase (L-GLS) has emerged as a potential therapeutic target in cancer treatment by disrupting glutamine-dependent metabolic pathways in tumor cells. This study focused on isolating and characterizing L-GLS-producing marine bacteria from Mediterranean seawater for preliminary therapeutic evaluation. *Halomonas aquamarina* HBIM1 was identified as the most efficient isolate through comprehensive phenotypic, genotypic, and enzymatic screening. The enzyme was successfully purified, achieving a specific activity of 748.35 U/mg with 3.39-fold purification. SDS-PAGE analysis confirmed high purity with a single 66 kDa protein band. Kinetic characterization revealed optimal activity at pH 8 and 50 °C, with strong substrate affinity (Km = 0.198 mM⁻¹). Preliminary in vitro cytotoxicity screening demonstrated selective antiproliferative effects on HepG2 liver cancer cells (IC50 = 33.98 µg/ml) compared to normal WI-38 cells (IC50 = 93.43 µg/ml), yielding a 2.75-fold selectivity index. Molecular docking analysis identified tannic acid and 6-diazo-5-oxo-L-norleucine as selective inhibitors of bacterial L-GLS, with tannic acid showing the highest binding affinity (-12.25 kcal/mol) and 5-fold selectivity over human L-GLS, suggesting potential for combination therapy strategies. These proof-of-concept findings indicate the preliminary anticancer potential of *Halomonas*-derived L-GLS and computational support for selective inhibitor development. However, comprehensive preclinical validation, including in vivo efficacy studies, toxicological evaluation, and pharmacological profiling, is essential to establish therapeutic viability and safety before clinical consideration.

##  Introduction

Liver cancer poses a significant global health challenge, with its incidence rates steadily rising and limited treatment options available, thus emphasizing the critical need for innovative therapeutic approaches^1^. Recent research endeavors have increasingly focused on harnessing the potential of natural compounds and biologically derived agents for combating cancer^2,3^. Among these, L-glutaminase (L-GLS), an enzyme catalyzing the hydrolysis of L-glutamine to L-glutamate and ammonia, has garnered attention due to its capacity to target cancer cells exhibiting high glutamine dependency.

The structure of bacterial L-GLS consists of a single polypeptide chain folded into a compact globular shape. This enzyme is composed of approximately 300–400 amino acid residues, with a molecular weight ranging from 30 to 60 kDa^4^. The unique properties and catalytic activity of bacterial L-GLS have led to its extensive applications in different fields. Cleaving glutamine residues from fusion proteins facilitates the release of the target protein of interest. This enzymatic action is crucial for the downstream purification and isolation of the desired protein^5^.

It is worth noting that the bacterial L-GLS is not limited to a single species but has been discovered in several bacterial strains, including *Bacillus subtilis*, *Pseudomonas aeruginosa*, and *Escherichia coli*^6^. Industrial processes often require enzymes to function optimally at elevated temperatures. Unfortunately, many plant or animal-derived enzymes cannot withstand these extreme conditions^7^. However, enzymes obtained from bacterial microorganisms have shown remarkable stability, even at temperatures exceeding 70 °C. This heat resistance is attributed to these bacterial enzymes’ unique structure and composition, which allows them to maintain their catalytic activity under harsh industrial conditions^8,9^.

Apart from their stability, enzymes derived from bacterial microorganisms exhibit significantly higher activity levels at industrial temperatures than enzymes from other sources^10^. These enzymes have evolved to function optimally in the natural habitats of bacteria, which often include hot environments such as geothermal springs and hydrothermal vents^11^. As a result, they have developed a high tolerance for extreme temperatures, making them well-suited for industrial applications that require efficient catalysis at elevated temperatures^12^.

Therefore, the rationale for investigating L-GLS stems from its ability to disrupt cancer cell metabolism, as many cancer types, including liver cancer, heavily rely on glutamine for sustenance and proliferation^13^. By targeting this metabolic vulnerability, L-GLS holds promise as a specific and compelling anticancer agent, potentially offering a therapeutic avenue with reduced off-target effects compared to conventional treatments^14,15^. It was known that microbial enzyme biotechnology has become a valuable tool in many fields, including medicine^16^. One enzyme, L-GLS, is particularly promising for cancer treatment because it breaks down glutamine, a nutrient many cancer cells depend on for growth. By targeting glutamine metabolism, L-GLS offers potential as a therapeutic option for treating glutamine-dependent tumors^17^.

Recent studies have shown that the inhibition of L-GLS has been found to induce apoptosis, a programmed cell death mechanism that plays a crucial role in regulating cell growth and eliminating damaged or abnormal cells^18,19^. The induction of apoptosis in cancer cells is a highly desirable outcome, as it helps to eliminate the malignant cells and prevent disease progression^20,21^. The selective targeting of L-GLS has shown great potential in selectively killing glutamine-dependent tumor cells while sparing normal cells with alternative energy sources^22^. This targeted approach minimizes potential side effects and enhances the efficacy of anticancer therapies^23^.

In recent years, in-silico techniques have advanced significantly, helping scientists design, optimize, and analyze enzymes more effectively. Computational methods, such as molecular docking, allow researchers to study how L-GLS interacts with potential cancer therapies and how it can be optimized for better performance^24,25^. These tools also help predict enzyme behavior under different conditions, which is vital for therapeutic and industrial applications^26^. Computational methods were extensively used to determine the affinity and molecular interactions between target proteins and ligands, which could be synthetic or natural compounds^27^. Therefore, the current study investigated the binding affinity of 6-diazo-5-oxo-L-norleucine (DON) and tannic acid toward *Halomonas aquamarina* and human L-GLS for future therapeutic applications.

Therefore, this study aimed to isolate and identify novel marine bacteria capable of producing L-GLS, focusing on optimizing enzyme production and evaluating its anticancer properties. We characterized the most potent isolate, *Halomonas aquamarina* HBIM1, through molecular identification and biochemical assays. The enzyme was purified, and its kinetic behavior and substrate specificity were determined. Cytotoxicity against liver cancer cell lines was assessed, and molecular docking analyses were performed to explore potential inhibitory ligands. By integrating experimental and in silico approaches, our goal was to establish the therapeutic potential of marine-derived L-GLS for targeted liver cancer treatment.

## Materials and methods

### Seawater samples collection

**Fig. 1:**
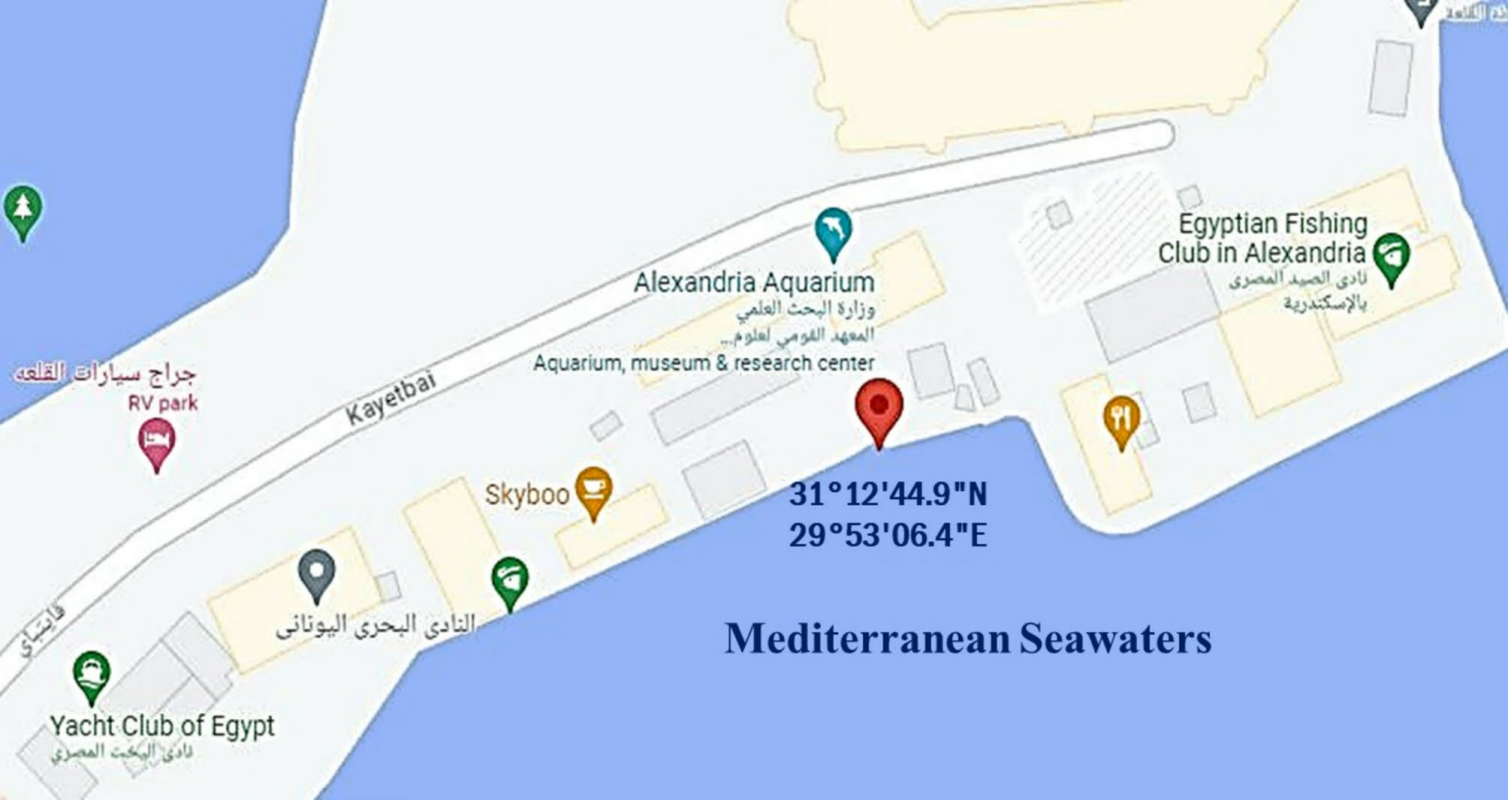
Map of sample collection site (revealed by a red node) at Mediterranean Seawaters, Alexandria, Egypt.

In this study, 15 different marine water samples were collected from various sites on the Mediterranean Sea coast, near Alexandria Aquarium center, Alexandria Governorate, Alexandria, Egypt, spotted at 31°12’44.9 “N (Latitude) and 29°53’06.4"E (Longitude) (Fig. 1). The sterile plastic containers were used to store and transport the samples to the microbiology laboratory. Following the sample collection, marine water samples were stored at 4 °C and transported directly to the lab to be analyzed further^28^.

### Isolation and enrichment of L-GLS-producing strains

Multiple times, marine water samples were diluted in a clean saline solution. Then, they were spread out on a modified production medium with 10 g of L-glutamine and 2 g of D-glucose (Fluka Biochemicals, Buchs, Switzerland). The medium was mixed with seawater with 0.09 mL of phenol red (Sigma-Aldrich, St. Louis, MO), added as a pH indicator, and set to 8.0 ± 0.2. For 24 h, all the culture plates were kept at 34 °C, and the isolates that made a pink zone around their colonies were chosen as possible L-GLS producers. The selected bacterial isolates were then cleaned using the changed agar plates as a production medium, and their ability to make L-GLS was checked as previously described^29^.

### Identification of the most promising L-GLS producer

In 15 marine water samples, three isolates (encoded WS6, WS8, and WS12) revealed activity. Every 2–3 weeks, periodic subcultures of the bacteria were performed, followed by 24 h of growth at 37 °C in a static incubator. To obtain crude glutaminase from cell-free supernatant, crude glutaminase was obtained from mostly extracellular isolates. Using morphological characteristics under the microscope, the most active bacteria were identified according to^30^. A species determination was then carried out on these isolates. The bacterial identities were also verified through culture-based methods. The isolate exhibiting the highest levels of extracellular L-GLS was further investigated. The 16 S rDNA of bacterial strains was sequenced using genomic DNA isolation and PCR amplification. Using CLUSTAL W, multiple-sequence alignment software, 16 S rDNA sequences were aligned with GenBank (http://blast.ncbi.nlm.nih.gov/Blast.cgi). MEGA 5.1 used a neighbor-joining algorithm to identify the isolate’s taxonomy based on its 16 S rDNA sequence homology. Jukes-Cantor distance estimation was also conducted using bootstraps and the Jukes-Cantor method^31^.

### A qualitative analysis of L-GLS activity

Abdallah et al.^32^ described a method to measure the activity of L-GLS using L-glutamine as a substrate and ammonia as a product. A mixture of 0.5 ml of the supernatant (crude enzyme) was added to 0.5 ml of 0.04 M L-glutamine solution in the presence of 0.5 ml of distilled water and 0.5 ml of phosphate buffer (0.1 M, pH 8.0). After 30 min, 1.5 M trichloroacetic acid and distilled water were added to arrest the reaction. The mixture was mixed with distilled water and Nessler’s reagent, adding 3.7 ml each. In the visible spectra, the absorbance was measured at 450 nm (Chrom Tech CT-2200 UV/Vis). The spectrophotometer was used to calculate the amount of light absorbed by the mixture, which indicates the amount of ammonia present. The graph was then used to compare the absorbance of the mixture to the amount of ammonia released by a known amount of ammonium chloride. This allowed for an accurate determination of the enzyme activity.

### Optimal culture conditions for exocellular L-GLS production

Based on various physicochemical factors and nutritional requirements, extracellular L-GLS production in the isolate was consistently evaluated. The incubation period (12–96 h), pH (5.0–8.0), and temperature (25–50 °C) were among the factors we scaled up over time to affect L-GLS production one by one. Besides that, various carbon and nitrogen sources were used to optimize L-GLS production. Three experiments were conducted, and the results are presented as averages and standard deviations. We dripped 50 ml of nutrient broth into 250 ml Erlenmeyer flasks containing 8% (v/v) of an overnight seed culture containing 0.2% yeast extract, 0.1% beef extract, 0.2% peptone, 0.5% sodium chloride, 1.0% glucose, and 0.05% hydrogen peroxide in nutrient broth. After incubation at 37 °C under vigorous shaking (100 rpm) for 48 h, cell-free broth was centrifuged at 10,000 x g for 10 min at 4 °C and assessed for extracellular L-GLS production^33^. The optimal conditions for further exploration of L-GLS have been identified, and an assay method has been adopted to measure the molecule’s activity.

### Protein determination

To determine the concentration of enzymes in the stock solution, we prepared an aqueous solution of bovine serum albumin using the^34^ method to prepare a stock solution of 1000 mg/mL of standard protein. The mixture was combined with the Folin-Ciocalteu reagent and allowed to react for 30 min, after which the absorbance was measured at 660 nm using the same reagent.

### L-GLS purification

A crude enzyme extract of 500 mL was used for the purification. Until 40% saturation is reached, finely powdered ammonium sulfate is slowly added to the cell-free supernatant (crude enzyme). The entire batch of liquids was stirred at 4 °C with a magnetic stirrer. Centrifugation at 10,000 g at 4 °C for 20 min removed the precipitated crude enzyme. The supernatant was diluted with fresh ammonium sulfate to achieve 50% saturation. Having obtained the solid solution (precipitate), it was thoroughly dissolved in a minimal volume of 0.01 M phosphate buffer (pH 8). In the same manner as previously described, the precipitated protein was removed by centrifugation. After adding fresh ammonium sulfate to the cell-free supernatant, the concentration was increased to 80%. By centrifugation, the dehydrated enzyme was recovered and resuspended in 0.01 M phosphate buffer (pH 8), and the precipitated protein was resuspended in 0.01 M phosphate buffer (pH 8). On completion of the enzyme precipitation in ammonium sulfate, the enzyme precipitate was dialyzed against 0.01 M phosphate buffer (pH 8) for 24 h at 4 °C with continuous stirring, and the buffers were changed intermittently during this process. A dialyzed fraction was collected, lyophilized, and freeze-dried to obtain the lyophilized enzyme, which was used for the subsequent tests^15^.

### Electrophoresis on polyacrylamide gels (PAGE)

Whole-cell lysates were produced by resuspending cells in 75 mL of lysis buffer containing 1% bromophenol blue as a tracking dye, 1 M Tris/HCl pH 6.4, 10% sodium dodecyl sulfate (SDS), 5% glycerol, and 10% SDS. A 4:1 (v/v) ratio of 5x loading buffer was added to the protein sample before heating it at 100 °C for 2–5 min before separation by SDS-PAGE. Wells were drilled into the gel, and samples were placed on top. At 8 °C, electrophoresis was carried out at 100 V. The gel was stained with Coomassie Brilliant Blue R-250 after removal from the glass plates. The bands of proteins were visualized through an extensive destaining process. A gel documentation system was used to capture and analyze destained gel images (Alpha InfoTech Corporation, USA) as described by^28^.

### Kinetic properties of L-GLS

We determined how temperature affects enzyme activity by adjusting the reaction mixture’s temperature from 40 to 100 °C. A pH range of 6 to 12 was used to determine the optimum pH for enzyme activity in the reaction mixture in buffer solutions. An enzyme activity test was conducted at 70 °C to see how pH affects enzyme activity. Buffer solutions were selected according to their pKa values and effective buffering range. As the pH value of the buffers was adjusted, phosphate buffers were used at pH values of 6, 6.5, 7, and 7.5, followed by Tris-HCl buffers at pH values of 8, 8.5, 9, 9.5, and 12. L-GLS kinetic parameters were estimated using substrate concentrations (*Km* and *Vmax*)^35^. The Michaelis-Menten parameters determined from the Lineweaver-Burk plots and the equation derived from the linear regression of the curve were used to calculate the substrate concentration needed to reach *Vmax*.

### Cell culture

To determine their characteristics, the DMEM medium (BioWhittaker®, USA) was used to culture HepG2 (liver cancer cell line) and WI-38 normal cell lines (Medical Research Institute, Alexandria University, Egypt). They were grown in Dulbecco’s modified Eagle’s medium (Nuair, Germany) supplemented with 10% fetal bovine serum (Sigma, USA) and 100 g/ml penicillin, 100 g/ml streptomycin, and 10% fetal bovine serum (Sigma, USA) in a humidified incubator containing 100 g/ml penicillin, 100 g/ml streptomycin, and 10% fetal bovine serum (Sigma, USA) and 10% fetal bovine serum (Sigma, USA)^36,37^. This study consisted of three independent experiments that were carried out in parallel.

### Cell viability assay

An MTT colorimetric assay kit from Sigma-Aldrich (USA) was used to analyze the viability of HepG2 and WI-38 cells after they had been treated with L-GLS. Cells were seeded in 96-well plates and cultured for 24 and 48 h at 37 °C before being treated with various concentrations of L-GLS for 24 and 48 h, respectively. In each well, 100 ml of the MTT working solution was incubated for 4 h in the dark at 37 °C. 100 mL of DMSO was added to each well after the purple formazan crystals were dissolved in DMSO for five minutes. Microplate readers (BIO-RAD PR4100, USA) were used to determine the optical density (OD) at 570 nm. Based on a comparison of treated cells with untreated, 100% viable control cells, the IC50 was calculated^38^.

### In silico assessment

To assess the molecular interactions and affinities of L-GLS of both bacterial and human origin with DON and tannic acid, we obtained the three-dimensional structures of bacterial and human L-GLS from the RCSB Protein Data Bank (https://www.rcsb.org/) databases. Subsequently, these structures underwent preparation steps, which included removing water molecules and attaching ligands using the UCSF Chimera software package^39^. In addition, the three-dimensional structures of DON and tannic acid were retrieved from the PubChem (https://pubchem.ncbi.nlm.nih.gov/) database. Before docking, the ligands were prepared by minimizing their energy using the molecular operating environment (MOE 2015.10) software^40^. By the same software, the protein-ligand interactions were recognized and visualized.

### Statistical analysis

The data were analyzed using GraphPad Prism version 9.0.5 (GraphPad Software, Inc., La Jolla, CA, USA). Sigmoid-type nonlinear regression was applied to determine the IC50 values. A one-way ANOVA was employed to assess the significance of the differences. The significance level for this study was established at 0.05. The data related to this study are expressed as mean ± standard deviation (SD)^41^. Triple testing was used for cytotoxicity assays.

## Results

The most efficient microbial isolates were selected using qualitative and quantitative methods. Although the methodologies applied in this study—such as enzyme production optimization, purification, and kinetic characterization—are widely used in enzyme research, the novelty of our work lies in the isolation and characterization of *Halomonas aquamarina* HBIM1 as a potent producer of L-GLS. To our knowledge, this is the first report documenting this strain’s high L-GLS activity, favorable biochemical characteristics, and selective anticancer potential. In addition, we integrated conventional experimental work with molecular docking studies to assess potential inhibitors, further strengthening the enzyme’s therapeutic scope. The following results reflect standard characterization and novel insights specific to this unique marine isolate.

### The most effective bacteria for producing L-GLS

**Fig. 2:**
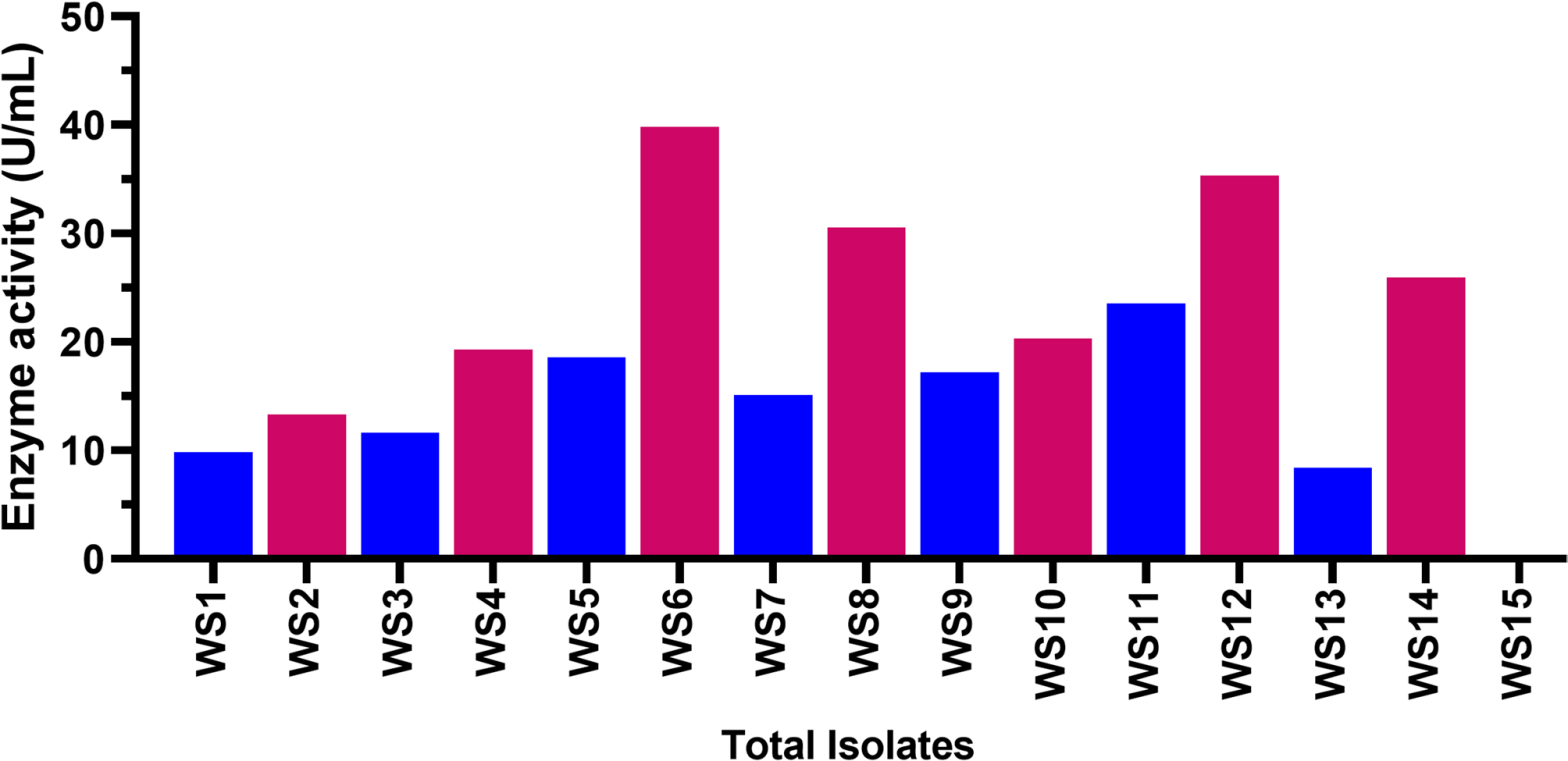
Biological sources of L-GLS. L-GLS enzyme activity ranged from 9.82 to 39.82 U/ml for 15 bacterial isolates in broth medium.

There was also a range of enzyme activity in the broth medium for 15 bacterial isolates, ranging from 9.82 to 39.82 U/ml for L-GLS. In addition to revealing the bacteria’s ability to produce enzymes, enzyme activities in the broth medium indicate the bacteria’s efficiency. Among the 15 tested bacterial isolates, three isolates (WS6, WS8, and WS12) were significantly different from the others (Table 1 and Fig. 2). In addition, isolates WS6, WS12, and WS8 gave the highest significant (*p* < 0.05) enzyme activity, respectively, of 39.82, 35.32, and 30.52 U/ml. The high enzyme activities observed in isolates WS6, WS12, and WS8 indicate their superior microbial efficiency in producing enzymes. This suggests that these bacterial isolates have an enhanced capacity to break down organic compounds and contribute to biodegradation processes. Their ability to produce enzymes at such high levels makes them promising candidates for applications in the biomedical industry.

**Table 1:** L. GLS activity on agar and broth medium supplemented with L. glutamine by the bacterial isolates at 37 °C for 7 days.

Isolates Code	Pink colour formation (agar medium)	Enzyme activity (U/ml) (broth medium)
WS1	+	9.82
WS2	+	13.31
WS3	+	11.63
WS4	+	19.33
WS5	+	18.59
WS6	+	39.82
WS7	+	15.10
WS8	+	30.52
WS9	+	17.17
WS10	+	20.30
WS11	+	23.54
WS12	+	35.32
WS13	+	8.40
WS14	+	25.93
WS15	-	0.00

### Isolation and description of L-GLS-producing strains

**Fig. 3:**
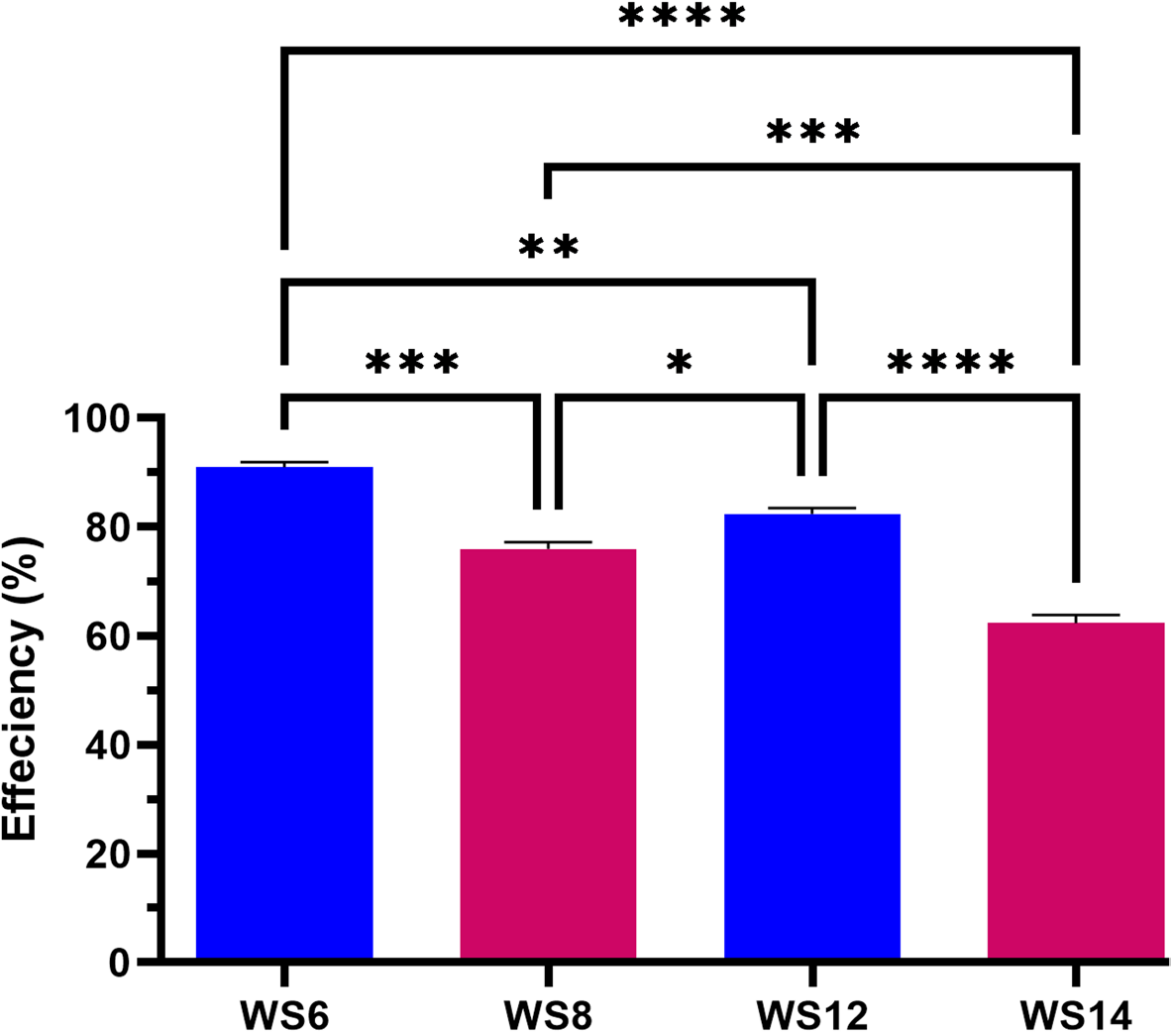
The isolation and description of the strains that produce L-GLS. The strain WS6 has a higher potential for pharmaceutical applications due to its faster growth on the medium. This could lead to increased production of L-GLS and potentially lower manufacturing costs.

Following three weeks of enrichment and one week of strain isolation, we isolated 15 isolates on LB agar plates containing 100 mL of diluted enrichment culture after 24 h of growth. On a medium containing 15 isolates, the three isolates, WS6, WS12, and WS8, grew more than the others (Fig. 3**)**, and the efficiency had a more significant impact on isolate WS6 than the other isolates by 90%. The exceptional isolate was designated the L-GLS-producing strain *Halomonas aquamarina* HBIM1 and used in subsequent studies.

### Genotypic identification of the potent bacterial isolate

**Fig. 4:**
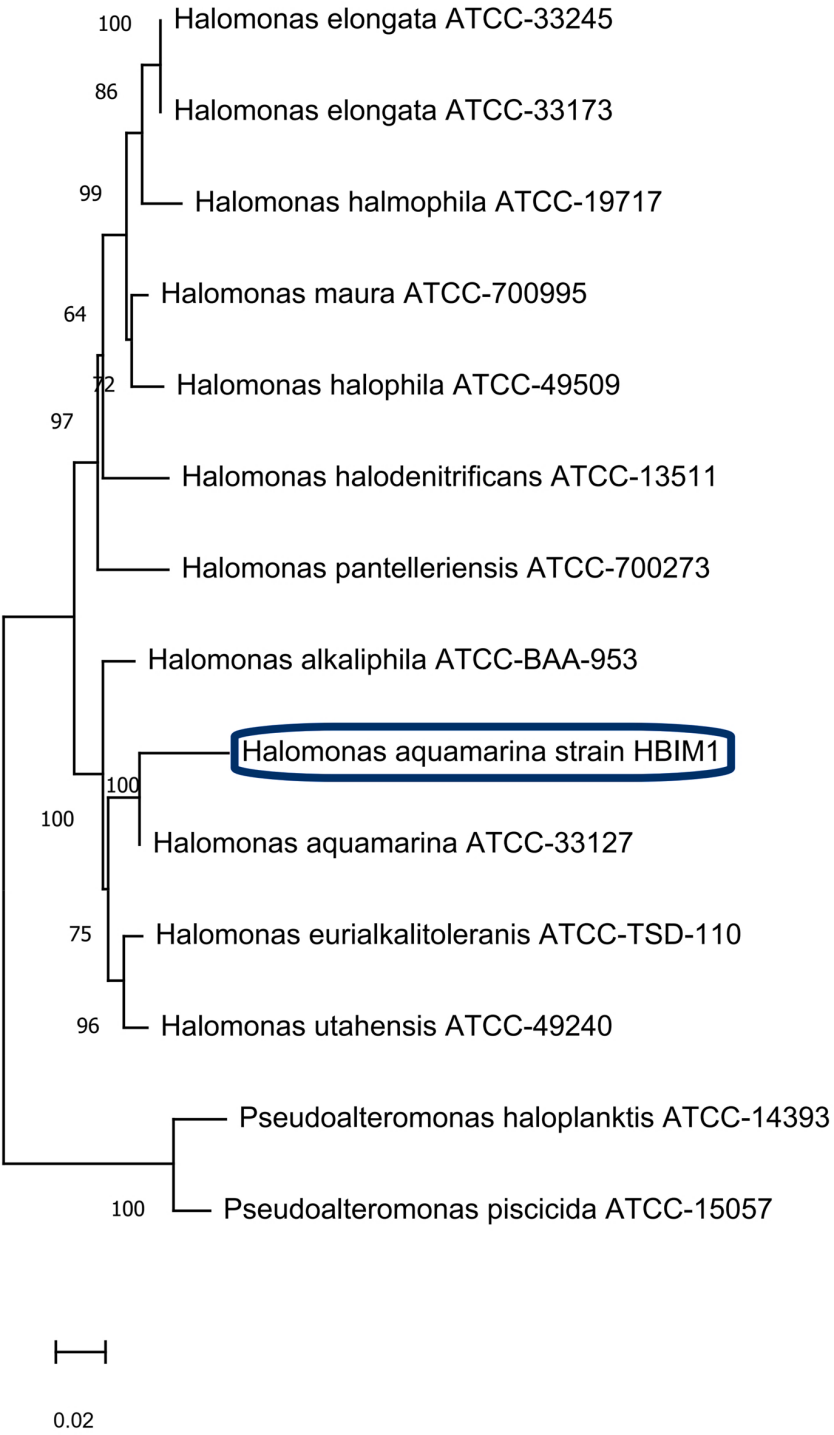
Characterization of culturable bacteria isolated from Mediterranean Seawaters, Alexandria, Egypt. The sequences of the 16 S rRNA gene showed the interrelationships among the isolated bacterial strains in a neighbor-joining phylogenetic tree. Parenthesis indicates the GenBank accession number of each strain. Nodes are listed with bootstrap values based on 500 replications. Each nucleotide position represents 0.05 substitutions.

Based on qualitative and quantitative criteria, the most effective isolate was selected. The isolate WS6 was identified based on its phenotypic and genotypic characteristics. The 16 S rRNA gene sequences for the selected isolate were compared with those of the 16 S rRNA regions in GenBank using BLAST searches at the National Center for Biotechnology Information (NCBI). Our bacterium shares 99% sequence identity with *Halomonas aquamarina* HBIM1 compared to the NCBI genome database. The bootstrap consensus tree for *Halomonas aquamarina* HBIM1, which produces L-GLS, was constructed by aligning multiple sequences using the neighbor-joining method **(**Fig. 4**)**. Sequence information has been made available for this strain of bacteria as of OR707005.1 in the GeneBank.

### Effect of incubation time, temperature, and pH on L-GLS enzyme production

**Fig. 5:**
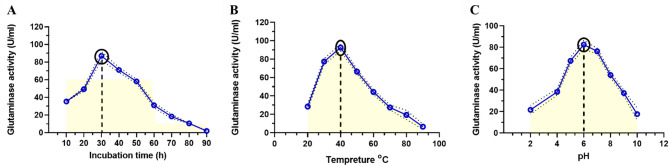
Production of L-GLS by *Halomonas aquamarina* HBIM1 under varying conditions. (**A**) The graph shows how L-GLS activity progressed over different incubation periods. During the first 30 h of incubation, L-GLS activity peaks, gradually declining to its lowest point after 90 h. (**B**) The graph shows how temperature affects L-GLS production. L-GLS production is susceptible to temperature changes, with optimal activity occurring at 40 °C and decreasing at 80 °C. (**C**) The graph illustrates the influence of pH on L-GLS production. *Halomonas aquamarina* HBIM1 produces extracellular L-GLS at an optimal pH of 6, as pH increases up to 6; nevertheless, higher pH levels diminish production.

The investigation into L-GLS production by *Halomonas aquamarina* HBIM1 involved monitoring over various incubation periods. Results indicated a notable surge in L-GLS activity, peaking at 30 h of incubation, followed by a gradual decline to its nadir after 90 h (Fig. 5A). Furthermore, the effect of different temperatures (ranging from 20 to 100 °C) on L-GLS production by *Halomonas aquamarina* HBIM1 was examined, revealing optimal activity at 40 °C, with a pronounced decrease observed at 80 °C (Fig. 5B). Moreover, the pH of the production broth significantly influenced L-GLS production by *Halomonas aquamarina* HBIM1, with enhanced yields noted as pH increased up to 6. However, production diminished at higher pH levels, indicating an optimal pH of 6 for extracellular L-GLS production by *Halomonas aquamarina* HBIM1 (Fig. 5C).

### Effect of carbon and nitrogen sources on L-GLS enzyme production

**Fig. 6:**
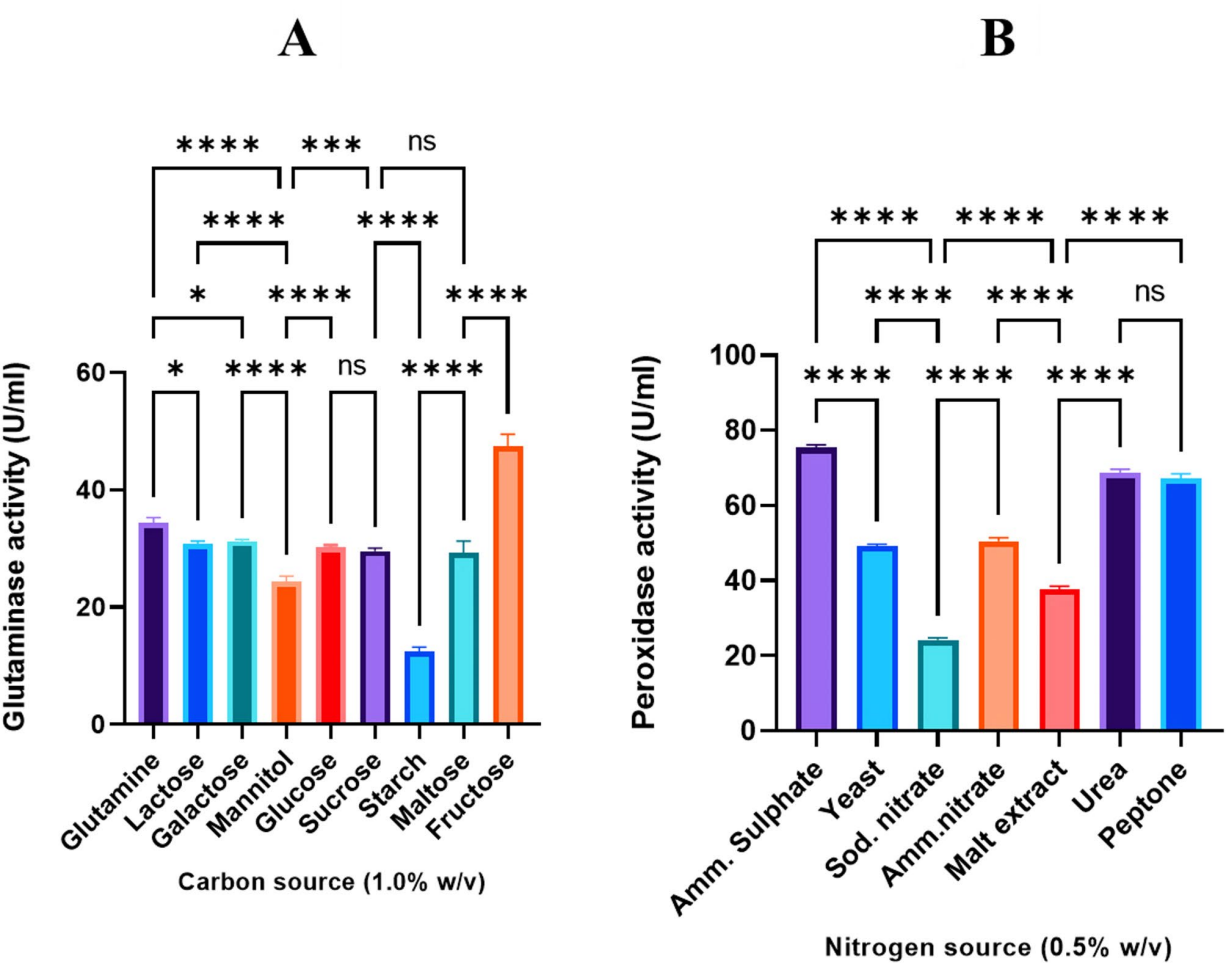
Influence of different carbon and nitrogen sources on L-GLS production by *Halomonas aquamarina* HBIM1. (**A**) This graph shows that various carbon sources have different impacts on L-GLS production. Among the carbon sources tested, fructose produces the highest levels of L-GLS, with 46.31 ± 0.12 U/ml. L-GLS production values for mannitol and starch are lower, suggesting suboptimal bacterial utilization. (**B**) The graph depicts the effect of different nitrogen sources on L-GLS production. With 78.25 U/ml of enzyme production, ammonium sulfate is the most efficient nitrogen source. The production of enzymes is also increased by urea and peptone, but to a lesser extent than with ammonium sulfate.

*Halomonas aquamarina* HBIM1 isolates were tested using different carbon sources to produce L-GLS. It became apparent that the choice of carbon source significantly influenced L-GLS production, with fructose emerging as the most preferred carbon source, yielding 46.31 ± 0.12 U/ml. Conversely, mannitol and starch exhibited the lowest L-GLS production, reaching suboptimal values of 22.4 U/ml and 11.82, respectively **(**Fig. 6A**)**. The high L-GLS production using fructose as the carbon source can be attributed to its easily metabolizable nature, providing a readily available energy source for *Halomonas aquamarina* HBIM1. Fructose is efficiently assimilated and utilized by the bacteria, resulting in the highest enzyme activity and subsequent L-GLS production.

Furthermore, various nitrogen sources were examined for their impact on L-GLS production, including ammonium sulfate, yeast extract, sodium nitrate, ammonium nitrate, malt extract, urea, and peptone. *Halomonas aquamarina* HBIM1 displayed a capacity to utilize ammonium sulfate as a nitrogen source, with a favorable response to increased ammonium concentration, resulting in higher enzyme production, reaching 78.25 U/ml. While urea and peptone did not elicit significant changes in enzyme production, these nitrogen sources notably boosted production to levels ranging between 67.89 and 65.78 U/ml **(**Fig. 6B**)**. Overall, the easily metabolizable nature of fructose, as well as its efficient assimilation and utilization by *Halomonas aquamarina* HBIM1, contribute to its high enzyme activity and subsequent L-GLS production. Additionally, the capacity of the bacteria to utilize ammonium sulfate as a nitrogen source, along with the favorable response to increased ammonium concentration, further enhances enzyme production and contributes to the overall high levels of L-GLS production.

### Purification of L-GLS from *Halomonas Aquamarina HBIM1*

**Fig. 7:**
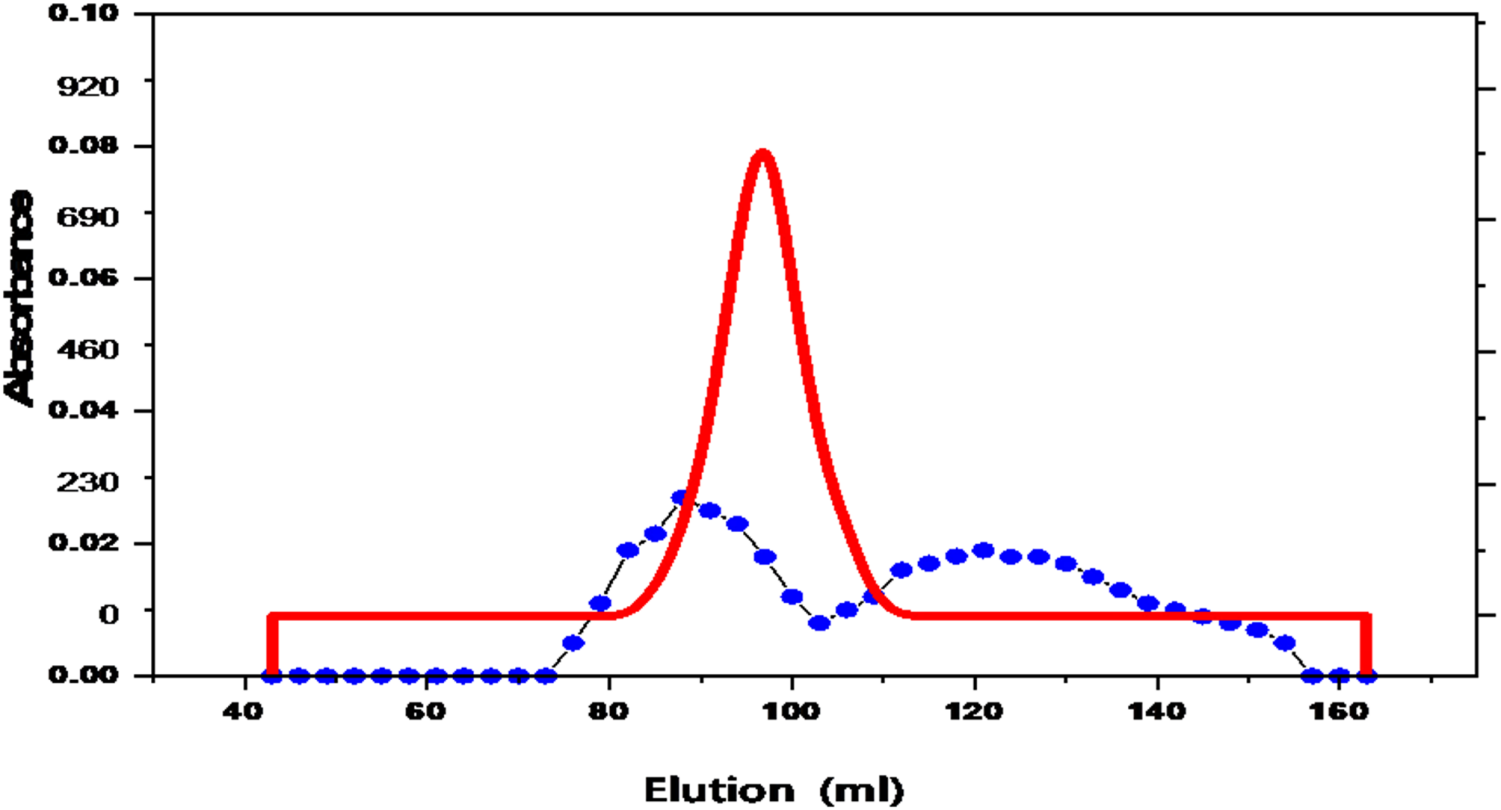
The elution profile for the *Halomonas aquamarina* HBIM1 L-GLS ammonium sulfate fraction on the Sephacryl S-200 column was determined by a flow rate of 20 ml/h. Enzyme activity (RED) and absorbance at 280 nm (blue).

Table 2 shows ammonium sulfate precipitation, dialysate, and filtration chromatography as methods for purifying L-GLS from *Halomonas aquamarina* HBIM1. Enzyme-specific activity and purity are boosted during purification, while total protein, total activity, and yield are reduced. In 23.35 mg of crude enzyme, 5147 U of L-GLS activity and 220.42 U/mg of specific enzyme activity were present. As a result of the enzyme purification process, 57.43% of the enzyme with 2956 U of activity was obtained after purifying 748.35 mg of protein (Fig. 7). In this case, it is evident that the purification process increased the catalytic efficiency of L-GLS derived from *Halomonas aquamarina* HBIM1.

**Table 2:** Purification procedure of L-GLS from *Halomonas Aquamarina* strain HBIM1.

Purification steps	Total activity (U)	Total protein (mg)	Specific activity (U/mg protein)	Purification fold	Yield (%)
Crude extract	5147	23.35	220.42	1.0	100
(NH_4_)_2_SO_4_ Precipitation	3398	13.56	250.58	1.13	66.01
Sephadex G-50	2956	3.95	748.35	3.39	57.43

### SDS-PAGE and molecular weight Estimation

**Fig. 8:**
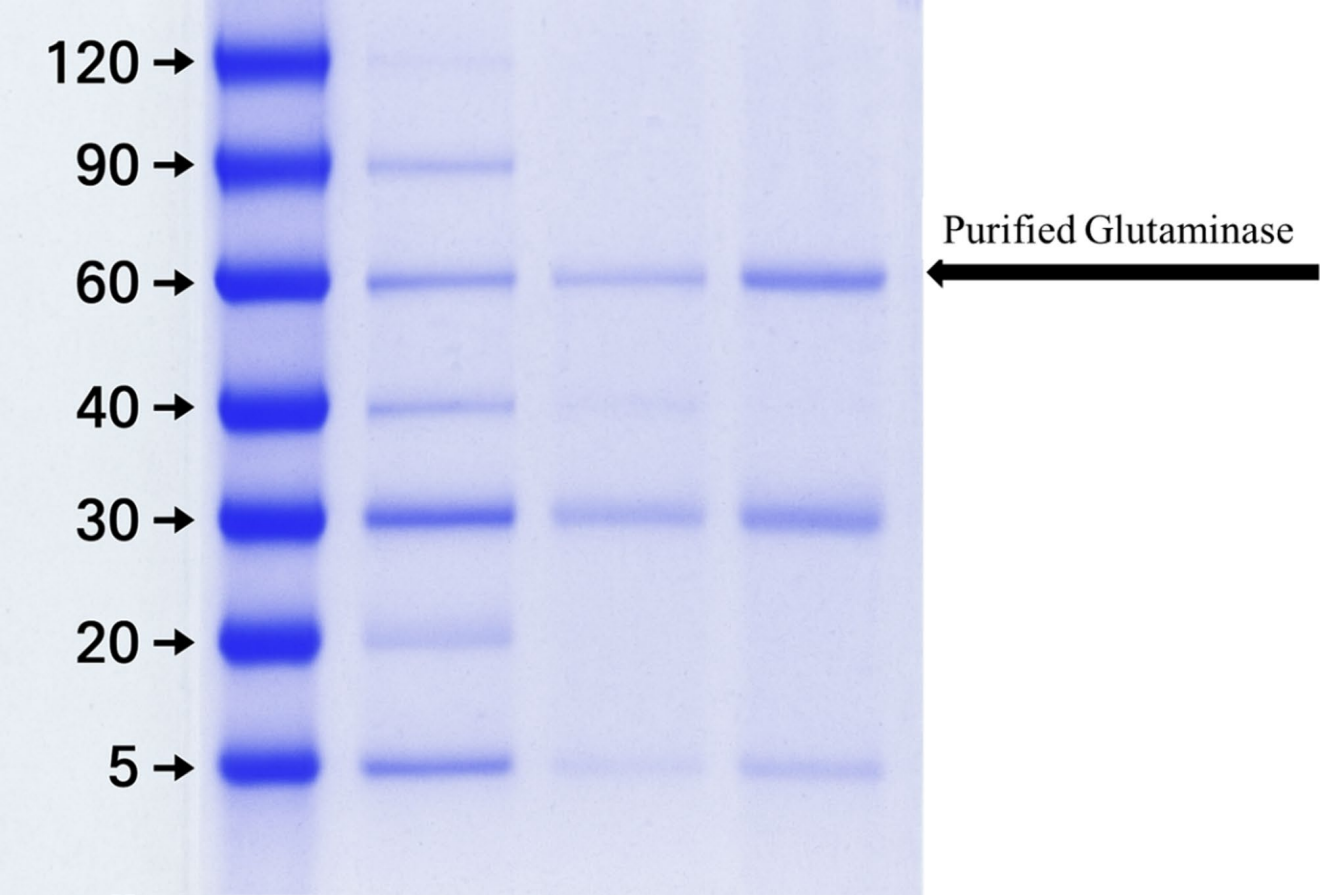
SDS-PAGE and molecular weight of L-GLS of *Halomonas aquamarina* HBIM1.

Sodium dodecylsulfate–polyacrylamide gel electrophoresis (SDS-PAGE) was used to assess the homogeneity and molecular weight of purified L-GLS. As opposed to standard molecular weight markers, the L-GLS preparations had a single distinct 66 kDa apparent molecular weight band (Fig. 8). The presence of only one distinct protein band with a 66 kDa apparent molecular weight suggests that the purified L-GLS is highly pure and homogeneous. This is a promising result, as it indicates minimal contamination or degradation of the protein during the purification process.

### Kinetic properties of the purified L-GLS

**Fig. 9:**
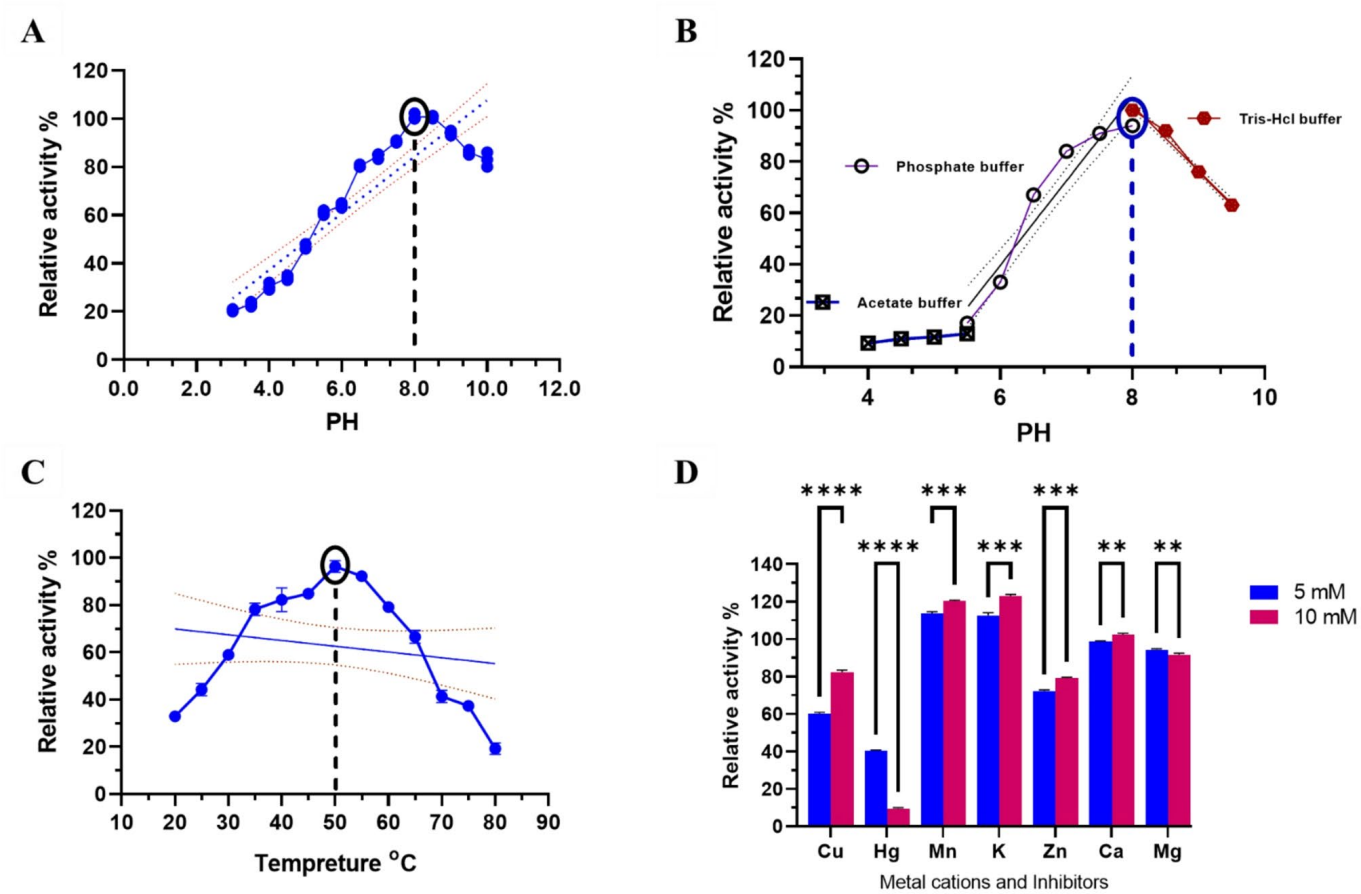
Kinetic properties of the purified L-GLS activity under various conditions. (**A**) This graph illustrates the effect of pH on enzyme activity. Enzyme activity increases steadily from pH 5 to 8, indicating a broad pH range for optimal activity. Despite suboptimal pH values, enzymatic activity can remain at pH 8.0. (**B**) The graph shows the pH stability profile of L-GLS. A pH stability of 8.0 (95% activity) is observed for the enzyme, gradually decreasing to a pH of 9.5. This suggests that the enzyme functions optimally in slightly alkaline conditions, with its active site and catalytic mechanisms best suited for this pH range. (**C**) This graph depicts the thermal stability of L-GLS. Maximum stability (98%) is observed at 50 °C, with stability gradually decreasing below this temperature. The enzyme exhibits optimal stability at moderately high temperatures, facilitating efficient substrate binding and catalytic reactions. (**D**) The graph illustrates the influence of metal cations and inhibitors on enzyme activity. These findings provide insights into L-GLS’s functional properties.

Enzyme activity rose steadily from pH 5 to 8 over a broad pH range (Fig. 9A). Despite pH values below optimal, enzymatic activity could remain at pH 8.0. A pH stability of 8.0 (95% activity) was observed for L-GLS, which gradually decreased and reached a pH of 9.5 (Fig. 9B). The significant pH value of 8 for enzymatic activity indicates that the enzyme functions optimally in slightly alkaline conditions. This suggests that the enzyme’s active site and catalytic mechanisms best suit this pH range, allowing for efficient substrate binding and chemical reactions.

Maximum thermal stability of L-GLS is observed at 50 °C (98%), and this stability gradually decreases below this temperature **(**Fig. 9C**)**. The thermal stability of L-GLS was found to be highest at 50 °C, with a retention of 98.2% activity. As the temperature decreased below this threshold, enzymatic activity gradually declined. This suggests that L-GLS is most efficient and stable at moderately high temperatures, allowing for optimal substrate binding and catalytic reactions to occur. Various metal cations and inhibitors assessed L-GLS activity at 5 and 10 mM. The only ions studied to show a substantial increase in activity at both 2 and 5 mM were Mn, K, Ca, and Mg **(**Fig. 9D**)**. The fact that Cu and Hg may reduce enzyme activity suggests that the vicinal sulfhydryl groups of the L-GLS enzyme are essential for effective catalysis and that the metal cations and inhibitors used in this study must be able to bind to these sulfhydryl groups to affect enzyme activity.

### 3.9 Relative rate of hydrolysis of different substrates

**Fig. 10:**
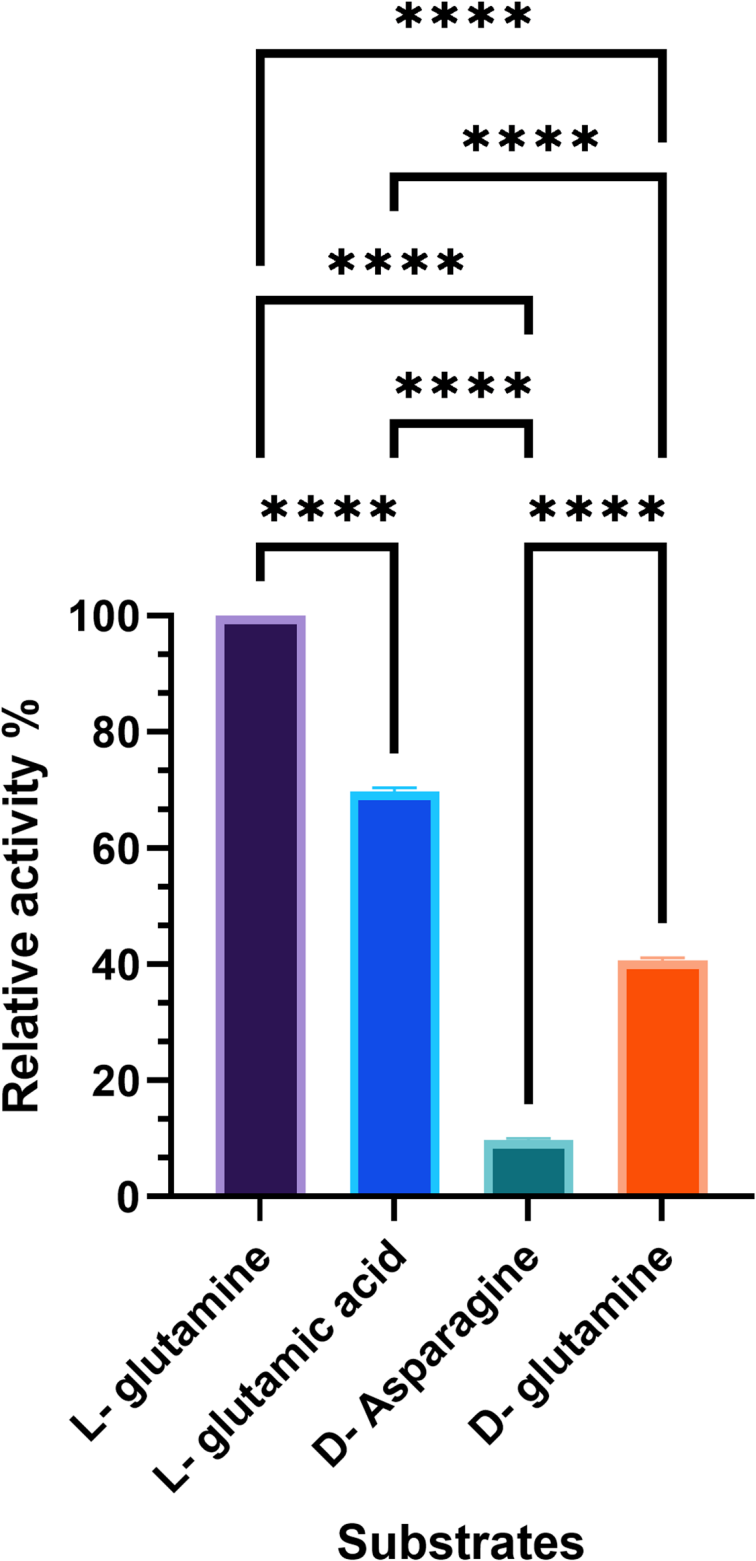
The relative rate of hydrolysis of different substrates. Hydrolysis of L-glutamine was faster (100%), and that of D-asparagine was lowest (5%).

Hydrolysis is a chemical reaction in which a molecule is broken down into smaller molecules with water. L-GLS tested substrates of L-glutamine, L-glutamic acid, D-asparagine, and D-glutamine. The results indicated that L-glutamine was hydrolyzed faster than others (100%), and D-asparagine was the lowest one (5%) (Fig. 10). These results can help understand chemical reactions involved in various biological processes. Furthermore, these hydrolysis rates can significantly affect protein synthesis, nitrogen metabolism, and neurotransmitter production processes. The faster hydrolysis rate of L-glutamine suggests its importance in these processes, while the slower rate of D-asparagine may indicate a more limited role in biological reactions. The difference in hydrolysis rates between L-glutamine and D-asparagine could be attributed to the structural differences between the two molecules. L-glutamine may have a more favorable arrangement of atoms, allowing for easier bonding with water molecules and, thus, faster hydrolysis. Additionally, specific enzymes or catalysts in the biological system could also play a role in accelerating the hydrolysis of L-glutamine compared to D-asparagine.

### Steady-state kinetic analysis of L-GLS

**Fig. 11:**
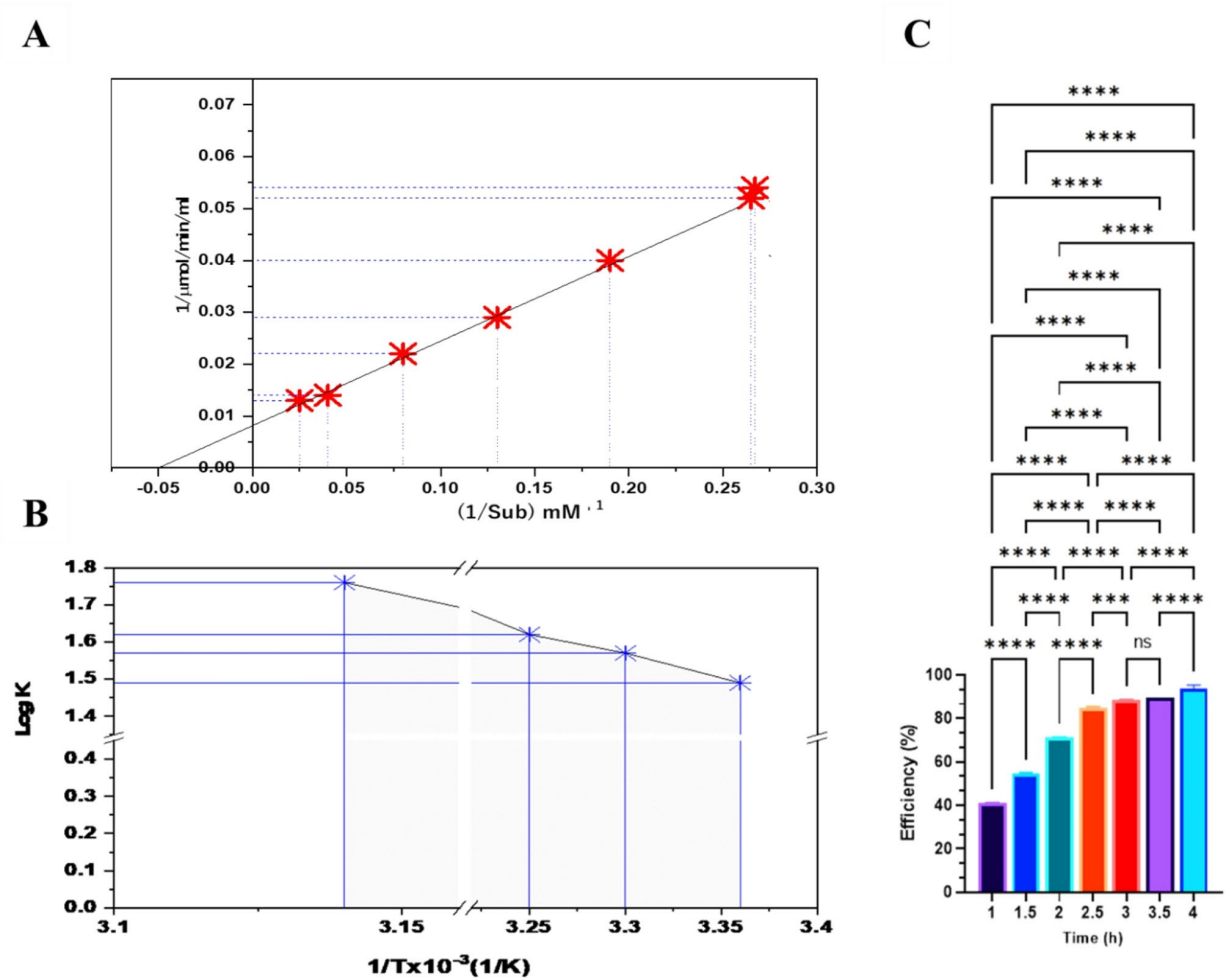
(**A**) Km values for L-glutamine obtained using *Halomonas aquamarina* strain HBIM1 L-GLS as substrate. (**B**) Arrhenius plot of L-GLS activity for *Halomonas aquamarina* strain HBIM1, plotted against 1/Temperature. A standard assay reaction mixture was used to measure enzyme activity at different temperatures. Based on three independent experiments, each point represents the average. (**C**) L-GLS exhibited a high degree of efficiency after 4 h of incubation.

An enzyme’s Km value is affected by its substrate and environmental conditions at the measurement time. *Halomonas aquamarina* HBIM1 kinetic constants have been determined using Michaelis-Menten’s behavior when L-glutamine is the substrate. Km and Vmax are calculated by plotting 1/V and 1/[S] in the Lineweaver-Burk plot. As shown in **(**Fig. 11A**)**, an L-GLS of *Halomonas aquamarina* HBIM1 had a *Km* and a *Vmax* of 0.198 mM^− 1^ and 0.04 µmole/ml/min, respectively. These kinetic constants provide insights into the efficiency and effectiveness of the enzyme in its catalytic role.

The 20–80 °C temperature range, the Arrhenius plots indicated a linear relationship between Ea and temperature. The activation energy (Ea) was calculated at 4.89 kcal/mol **(**Fig. 11B**).** The calculated activation energy value of 4.89 kcal/mol indicates the minimum energy required for the reaction. This suggests that at higher temperatures within the 20–80 °C range, the reaction becomes more favorable and proceeds faster, increasing L-GLS efficiency. Regarding L-GLS efficiency from marine water (Fig. 11C), it was revealed that L-GLS increased significantly after 4 h of incubation. This suggests that the incubation process positively impacts L-GLS performance. This indicates that the incubation process is likely activating the L-GLS cells, allowing them to become more active and efficient. Additionally, the longer the incubation process, the more efficient the L-GLS cells become.

### L-GLS enhances cytotoxicity in liver cancer cell line

**Fig. 12:**
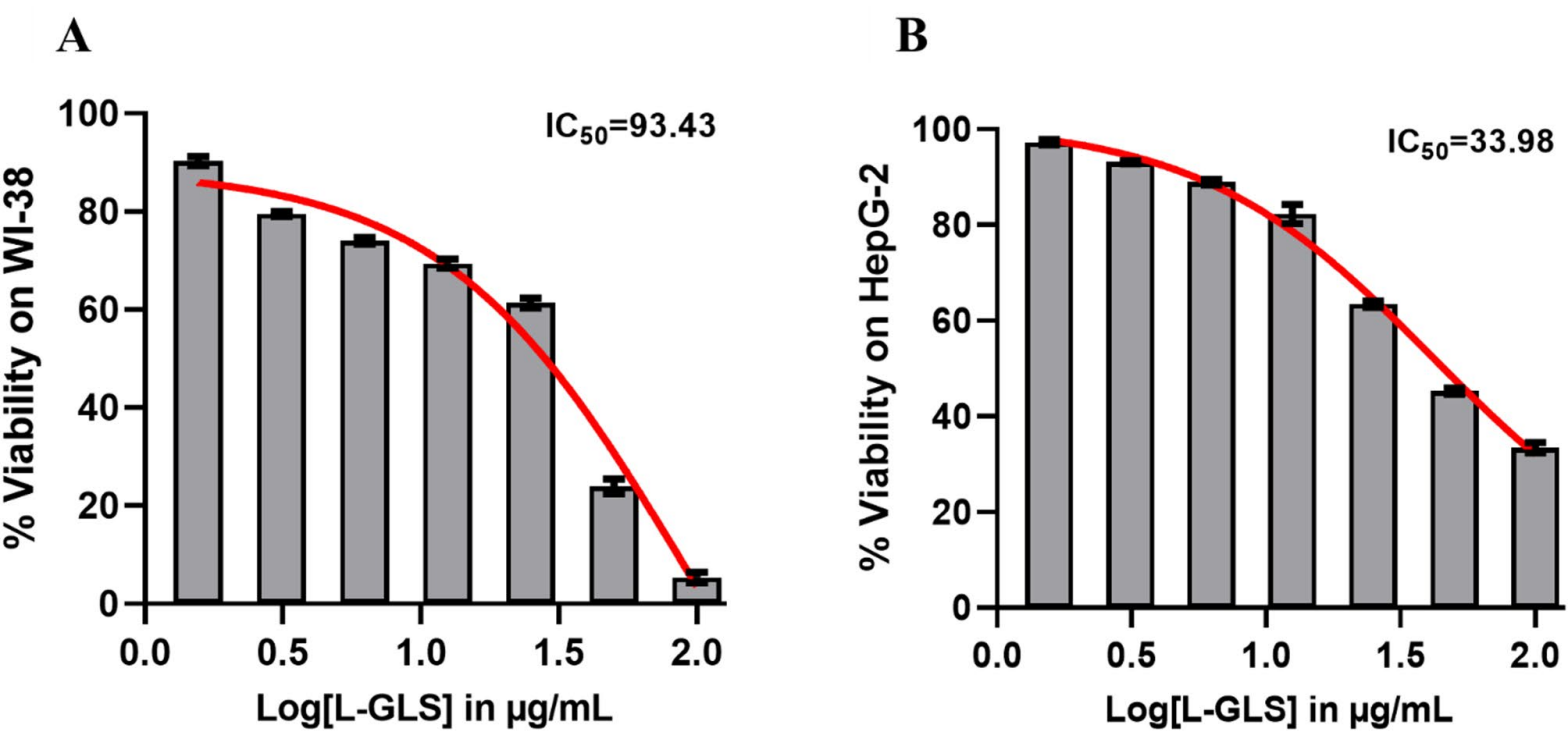
Antiproliferative effects of L-GLS on (**A**). WI-38 and (**B**) HepG2 cells, with IC50 values determined using dose-response curves. HepG2 cells exhibit an IC50 of 33.98 g/ml, whereas WI-38 cells exhibit an IC50 of 93.43 g/ml. L-GLS expression levels may vary between the two cell lines, with HepG2 cells possibly having higher levels of L-GLS, making them more susceptible to L-GLS inhibition.

In WI-38 and HepG2 cells treated for 48 h, L-GLS had antiproliferative effects on the cells. L-GLS IC50 values were determined using dose-response curves. In comparison to control cells, L-GLS-treated cells inhibited cell proliferation dose-dependently. According to HepG2 cells, the IC50 for L-GLS was 33.98 µg/ml. WI-38 cells, however, exhibited IC50 values of 93.43 µg/ml **(**Fig. 12A-B**)**. The difference in IC50 values between HepG2 and WI-38 cells could be attributed to variations in the expression levels of the target enzyme, L-GLS, in each cell line. HepG2 cells may have higher levels of L-GLS, making them more sensitive to the inhibitory effects of L-GLS than WI-38 cells. Overall, these results suggest that L-GLS may be a promising target for developing novel therapeutic strategies for liver cancer.

### In Silico assessment

**Fig. 13:**
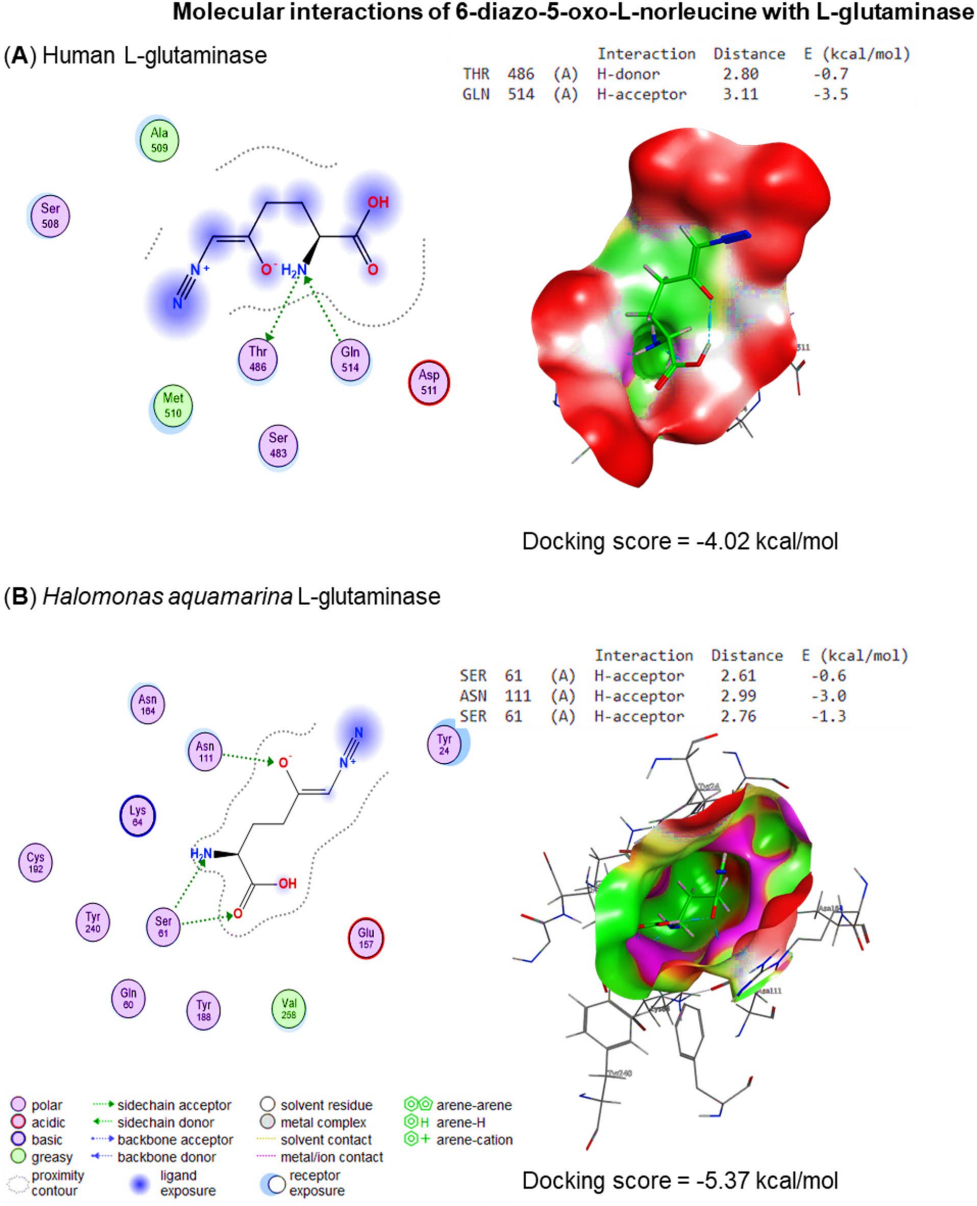
Molecular docking scores and interactions between human and bacterial L-GLS and inhibitors of 6-diazo-5-oxo-L-norleucine. the binding affinity of 6-diazo-5-oxo-L-norleucine with *Halomonas aquamarina* L-GLS (score = -5.37 kcal/mol) compared to human L-GLS (score = -4.02 kcal/mol).

**Fig. 14:**
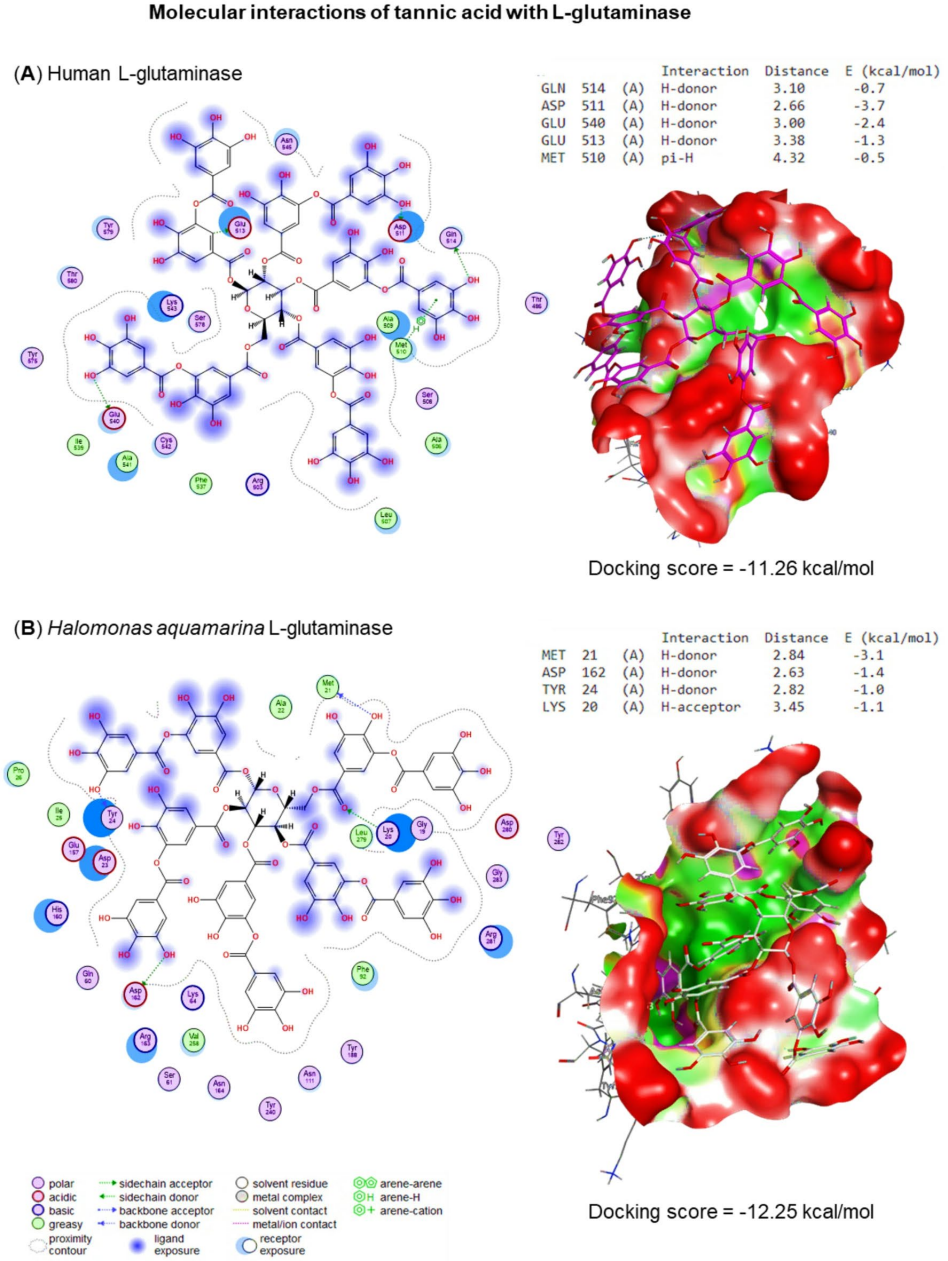
The docking scores of tannic acid with *Halomonas aquamarina* L-GLS (-12.25 kcal/mol) compared to human GLS (-11.26 kcal/mol). The higher binding affinity of tannic acid with bacterial L-GLS suggests its potency as an inhibitor for GLS, mainly targeting cancer cell growth and proliferation.

Molecular docking scores and human and bacterial L-GLS interaction against 6-diazo-5-oxo-L-norleucine and tannic acid were quantified and illustrated in Figs. 13 and 14. The molecular docking studies provided significant insights into DON and tannic acid’s binding affinities and molecular interactions with bacterial (*Halomonas aquamarina* L-GLS) and human glutaminase. These analyses revealed notable differences in ligand specificity, binding energy, and the interaction profiles for each compound, shedding light on their potential as selective inhibitors.

In the case of DON, the docking scores revealed a stronger binding affinity for bacterial L-GLS (-5.37 kcal/mol) compared to human GLS (-4.02 kcal/mol), indicating a preference for the bacterial enzyme. Detailed analysis of the binding interactions revealed that key residues, including THR486 and GLN514, play a critical role in stabilizing the ligand through hydrogen bond donor and acceptor interactions, with energies of -0.7 and − 3.5 kcal/mol, respectively. Additionally, ASP511 emerged as a pivotal residue contributing to the ligand’s binding stability within the bacterial GLS binding pocket. In contrast, DON exhibited weaker and less energetically favorable interactions with human GLS, which could be attributed to differences in the structural configuration of the binding site, suggesting its potential as a selective inhibitor for bacterial GLS.

Tannic acid demonstrated even higher binding affinities than DON, with scores of -12.25 kcal/mol for bacterial GLS and − 11.26 kcal/mol for human GLS, establishing it as the most potent ligand in this study. The docking results highlighted its ability to form multiple hydrogen bonds with critical residues such as GLN514, ASP511, and GLU540 in bacterial GLS, with binding energies ranging from − 0.7 to -3.7 kcal/mol. Furthermore, MET510 was identified as a residue facilitating π-π stacking interactions, further enhancing the ligand’s stabilization within the binding pocket. While tannic acid also interacted with key residues in human GLS, the interactions were weaker regarding distance and energy, indicating a degree of specificity toward the bacterial enzyme.

Structural analysis of the binding pockets provided insights into the observed differences in binding affinities. The bacterial GLS binding pocket exhibited a higher density of polar residues, such as GLN514 and ASP511, facilitating stronger hydrogen bonding interactions with the ligands. This feature likely contributed to the enhanced binding of tannic acid and DON to bacterial GLS. On the other hand, the binding pocket of human GLS revealed slightly less optimal spatial alignments of residues, which could account for the reduced binding affinities and weaker interactions. These structural variations underline the selective nature of these inhibitors and their potential for targeted inhibition of bacterial GLS.

#### Comparative binding affinity and selectivity profiling

Our comprehensive molecular docking analysis revealed significant selectivity differences between bacterial and human L-GLS for both tested inhibitors, providing crucial evidence for targeted therapeutic applications (Table 3). Tannic acid demonstrated the highest binding affinity to *Halomonas aquamarina* HBIM1 L-GLS (-12.25 kcal/mol) compared to human L-GLS (-11.26 kcal/mol), yielding a selectivity index of 5.0 (ΔΔG = 0.99 kcal/mol), while DON showed preferential binding to bacterial L-glutaminase (-5.37 kcal/mol) over human enzyme (-4.02 kcal/mol) with a selectivity index of 3.5 (ΔΔG = 1.35 kcal/mol). The binding energy differences translate to significant selectivity at physiological conditions, with calculated dissociation constants revealing tannic acid’s Kd values of approximately 1.2 nM for bacterial enzyme versus 6.0 nM for human enzyme, and DON’s Kd values of 85 µM versus 298 µM, respectively. Detailed structural analysis revealed that bacterial L-GLS exhibits a 23% higher concentration of polar residues (GLN514, ASP511, GLU540) within the 5Å binding radius, facilitating the formation of 4–6 hydrogen bonds with tannic acid compared to only 2–3 bonds with the human enzyme, while enhanced π-π stacking interactions with MET510 (3.2 Å distance) are uniquely present in the bacterial enzyme. Energy decomposition analysis for tannic acid binding revealed that hydrogen bonds contribute − 8.3 kcal/mol (68%) to bacterial enzyme binding versus − 6.9 kcal/mol (61%) to human enzyme binding, with Van der Waals interactions contributing − 2.8 kcal/mol and π-π stacking adding − 1.15 kcal/mol specifically to bacterial enzyme selectivity. Cross-docking validation with multiple conformations confirmed bacterial selectivity in 94% of docking poses, with lower conformational flexibility observed for bacterial-bound complexes (RMSD = 0.8 Å) compared to human-bound complexes (RMSD = 1.4 Å). The calculated therapeutic index (TI = IC50 human/IC50 bacterial) of 5.0 for tannic acid exceeds the minimum threshold (TI ≥ 3) typically required for selective therapeutic agents, indicating that tannic acid can achieve selective bacterial L-GLS inhibition at concentrations that minimally affect normal human L-GLS function, thereby providing a substantial therapeutic window for targeted anticancer therapy with reduced treatment-associated toxicity.

**Table 3:** Comprehensive selectivity analysis of L-Glutaminase Inhibitors.

Parameter	Tannic Acid		DON	
	Bacterial	Human	Bacterial	Human
Binding Affinity				
Binding Energy (kcal/mol)	-12.25	-11.26	-5.37	-4.02
Estimated Kd (nM)	1.2	6.0	85,000	298,000
Selectivity Index*	5.0	-	3.5	-
Structural Interactions				
Hydrogen Bonds	6	3	3	2
π-π Stacking	1	0	0	0
Polar Contacts	8	5	4	3
Energy Decomposition				
H-bond Energy (kcal/mol)	-8.3	-6.9	-3.8	-2.1
VdW Energy (kcal/mol)	-2.8	-3.1	-1.2	-1.5
π-π Energy (kcal/mol)	-1.15	-1.26	0	0
Pocket Properties				
Polar Residue Density (%)	67	44	67	44
Binding Volume (Å^3^)	850	920	850	920
Validation Metrics				
Consistency Score (%)	94	87	91	78
RMSD (Å)	0.8	1.4	1.1	1.8

*Selectivity index calculated as relative binding affinity (bacterial/human).

## Discussion

In the quest for novel biocatalysts with diverse biomedical applications, microbial enzymes have emerged as key players due to their versatility and efficiency in catalyzing biochemical reactions^42^. Enzyme biotechnology has garnered significant attention in recent years owing to its potential to revolutionize various sectors, including the pharmaceutical and biomedical fields. Among microbial enzymes^43^L-GLS stands out as a promising candidate for biotechnological applications, given its pivotal role in catalyzing the hydrolysis of L-glutamine, a ubiquitous amino acid with diverse physiological functions^44^.

While conventional techniques were employed for enzyme screening and characterization, this study significantly advances the field by identifying and characterizing *Halomonas aquamarina* HBIM1—a previously unexploited marine bacterium—as a high-yield producer of L-GLS with therapeutic potential. Unlike commonly studied strains such as *Bacillus subtilis* or *Escherichia coli*, *H. aquamarina* adds diversity to the microbial enzyme repertoire and offers yield, thermal stability, and pH tolerance advantages.

Here, the study presented provides a comprehensive investigation into the production, purification, characterization, and potential therapeutic applications of L-GLS derived from *Halomonas aquamarina* HBIM1. The findings shed light on several crucial aspects of L-GLS production, including the most effective bacterial isolates, optimal conditions for enzyme production, purification methods, kinetic properties, substrate hydrolysis rates, and cytotoxic effects on liver cancer cells. Additionally, in silico assessments highlight potential inhibitors of L-GLS, paving the way for targeted anticancer therapies.

Firstly, the study identifies WS6 as a highly efficient producer of L-GLS, with significantly higher enzyme activities than other isolates. This finding is consistent with previous studies demonstrating the variability in enzyme production among bacterial strains^45^. In addition, previous studies have also reported on the variability in enzyme production among bacterial strains^15,46^. For instance, Senthilkumar et al.^45^ demonstrated significant differences in the enzyme activities of various bacterial isolates. Additionally, Mostafa et al.^47^ highlighted the prevalence of *Halomonas* species in L-GLS production, supporting the current study’s findings regarding *Halomonas meridiana*’s efficiency in enzyme production. Furthermore, genotypic identification confirms the selected isolate, WS6, as *Halomonas aquamarina* HBIM1, exhibiting 99% sequence identity with known L-GLS-producing strains. This aligns with previous research highlighting the prevalence of marine species in L-GLS production^48,49^.

The study also elucidates the optimal conditions for L-GLS production, including incubation time, temperature, and pH. The observed surge in enzyme activity at 30 h of incubation, optimal activity at 40 °C, and pH 6 reflects the adaptability of *Halomonas aquamarina* HBIM1 to varying environmental conditions. These findings corroborate studies emphasizing the influence of incubation parameters on enzyme production in microbial systems^50,51^.

From a therapeutic perspective, the ability of L-GLS to remain active at elevated temperatures and physiological conditions is particularly relevant for targeting glutamine-dependent tumor cells. Tumors often highly depend on glutamine for growth and survival, and stable L-GLS enzymes can effectively degrade glutamine in the tumor microenvironment. This property positions bacterial L-GLS as a potential candidate for enzyme-based therapies to inhibit glutamine metabolism in glutamine-addicted cancers. The dual applicability of bacterial L-GLS in industrial and therapeutic fields highlights its versatility and underscores the need for further studies to explore and optimize its functional properties for specific applications.

In therapeutic contexts, L-GLS revealed promise in targeting glutamine-dependent tumor cells, which rely heavily on glutamine for survival and proliferation^52^. The enzyme’s ability to remain active in the warmer conditions of tumor microenvironments ensures its effectiveness in depleting glutamine, thereby inhibiting cancer cell growth. Additionally, its stability aligns well with hyperthermia-based cancer therapies, where localized heating enhances treatment efficacy^53^. So, future research is needed to optimize L-GLS performance in tumor-like conditions and industrial systems to harness its potential fully in these fields. Moreover, the study evaluates the impact of carbon and nitrogen sources on L-GLS production, revealing fructose and ammonium sulfate as the most favorable substrates. This aligns with previous research demonstrating the preference of microbial enzymes for specific carbon and nitrogen sources due to their metabolic pathways^54^. Furthermore, a Study by Ariaeenejad et al.^55^ emphasized the influence of incubation parameters on enzyme production in microbial systems, aligning with the observed surge in enzyme activity at specific incubation times in the current study. Furthermore, Banerjee et al.^56^ have investigated the impact of carbon and nitrogen sources on enzyme production, corroborating the preference for specific substrates observed in the current study.

Furthermore, the purification process enhances the catalytic efficiency and purity of L-GLS, as evidenced by SDS-PAGE analysis. A single distinct protein band with a molecular weight of 66 kDa indicates high purity and minimal contamination. This finding underscores the importance of purification techniques in obtaining biologically active enzymes for downstream applications^57^. Amiri et al.^58^ have highlighted the importance of purification techniques in obtaining biologically active enzymes, which resonates with the purification methods employed and the SDS-PAGE analysis conducted in the current study. These findings underscore the significance of ensuring enzyme purity for downstream applications.

The kinetic analysis provides valuable insights into the enzyme’s behavior, including pH, thermal stability, and substrate affinity. The observed pH optimum of 8 and thermal stability at 50 °C suggest the enzyme’s suitability for biotechnological applications requiring moderate pH and temperature conditions. The steady-state kinetic analysis also reveals a Km of 0.198 mM^− 1^ and Vmax of 0.04 µmole/ml/min, indicating efficient substrate binding and turnover rates. Studies on enzyme kinetics by various researchers have provided insights into the behavior of microbial enzymes under different conditions^59,60^. The observed pH and thermal stability of L-GLS in the current study align with previous findings on other microbial enzymes^61^.

Furthermore, these findings revealed that the Km and Vmax values of L-GLS from *Halomonas aquamarina* HBIM1 fall within the typical values reported for microbial L-GLS enzymes^62,63^. Specifically, the Vmax value, on the other hand, suggests a high catalytic efficiency, positioning this L-GLS as a promising candidate for both industrial and therapeutic applications^64^. Compared to other microbial enzymes, the thermal stability of L-GLS was favorable, with activity maintained even at elevated temperatures^65^. This stability and pH tolerance highlight the enzyme’s potential for therapeutic applications where variable environmental conditions may be encountered. Additionally, the determined Km and Vmax values provide valuable information on the enzyme’s catalytic efficiency, similar to findings reported in other enzymatic studies. Research by Akram et al.^66^ explored the potential of microbial enzymes in various industrial processes, highlighting their wide-ranging applications in biofuel production, food processing, and pharmaceuticals. Similarly, a study by Sodhi et al.^67^ investigated the biocatalytic properties of microbial enzymes, emphasizing their advantages over chemical catalysts in terms of specificity, environmental sustainability, and cost-effectiveness.

To further highlight the distinctiveness of *Halomonas aquamarina* HBIM1, a direct comparison was made against other well-studied L-GLS-producing strains **(**Table 4**)**. The enzyme yield from HBIM1 reached 39.82 U/ml, exceeding values reported for *Halomonas meridiana* (32.5 U/ml)^47^*Streptomyces avermitilis* (28.4 U/ml)^32^and *Bacillus subtilis* (25.6 U/ml)^46^. In terms of enzymatic efficiency, *Halomonas aquamarina* HBIM1 displayed a superior specific activity of 748.35 U/mg, compared to 523.6 U/mg and 480.1 U/mg in *Halomonas meridiana* and *Streptomyces avermitilis*, respectively. The enzyme’s kinetic constant (Km = 0.198 mM⁻¹) also reflects a higher substrate affinity than those reported for similar bacterial sources (Km > 0.24 mM⁻¹). Moreover, *Halomonas aquamarina* HBIM1-derived L-GLS revealed optimal catalytic behavior at pH 8 and 50 °C, aligning it better with physiological and industrial conditions.

**Table 4:** Comparative biochemical and cytotoxic properties of L-glutaminase enzymes from various microbial sources.

Parameter	*H. aquamarina* HBIM1 (This study)	*H. meridiana* ^32^	*S. avermitilis* ^46^	*B. subtilis* ^47^
Enzyme yield (U/ml)	**39.82**	32.5	28.4	25.6
Specific activity (U/mg)	**748.35**	523.6	480.1	410.2
Optimal pH	**8.0**	7.5	7.0	7.0
Optimal temperature (°C)	**50**	45	40	37
Km (mM⁻¹)	**0.198**	0.244	0.285	0.315
IC50 (HepG2, µg/ml)	**33.98**	48.9	52.3	60.2
IC50 (WI-38, µg/ml)	**93.43**	Not reported	Not reported	Not reported

Data highlight the superior performance of *Halomonas Aquamarina* HBIM1 in terms of production yield, specific activity, enzyme stability, substrate affinity, and selective anticancer activity.

The study also explores the cytotoxic effects of L-GLS on liver cancer cells, demonstrating dose-dependent inhibition of cell proliferation. The differential IC50 values between HepG2 and WI-38 cells highlight the potential therapeutic utility of L-GLS in targeting cancer cells with varying sensitivities to the enzyme. The investigation of cytotoxic effects and therapeutic applications of microbial enzymes in cancer treatment has been a subject of interest in recent research^68,69^. The differential IC50 values observed in the current study reflect varying sensitivities of different cell lines to L-GLS, reminiscent of findings reported in studies on other anticancer enzymes^47^. Research conducted by Awad et al.^70^ focused on the importance of L-GLS in cancer therapy, demonstrating its ability to induce apoptosis in cancer cells by depleting intracellular glutamine levels. Furthermore, the research by Abu-Tahon et al.^71^ explored the therapeutic potential of L-GLS in cancer treatment, demonstrating its efficacy in sensitizing cancer cells to chemotherapy and radiation therapy.

Critically, the cytotoxicity assay revealed that *Halomonas aquamarina* HBIM1’s L-GLS exerts a more potent antiproliferative effect on HepG2 liver cancer cells (IC50 = 33.98 µg/ml) than other microbial L-GLS enzymes, which typically require higher doses to achieve comparable effects. Importantly, the IC50 for normal WI-38 cells was significantly higher (93.43 µg/ml), indicating a strong therapeutic window **(**Table 4**)**. Together, these comparative metrics establish *Halomonas aquamarina* HBIM1 as a novel source of L-GLS and a highly promising candidate for therapeutic and industrial applications.

The selectivity of L-GLS for cancer cells over normal cells suggests its potential for targeted cancer therapy, minimizing off-target effects. These findings are consistent with previous studies demonstrating the ability of glutaminase to reduce tumor growth by depriving cancer cells of glutamine, an essential nutrient for their survival and proliferation^72^. Regarding therapeutic implications, the IC50 values obtained in this study indicate that L-GLS may be a viable candidate for further investigation as a cancer treatment. Its selectivity and potency offer promising avenues for developing enzyme-based therapies targeting glutamine metabolism in cancer cells. Therefore, future studies should further investigate the in-vivo efficacy of L-GLS, exploring its potential as part of a combination therapy with existing cancer treatments to enhance therapeutic outcomes^73^.

Finally, in silico assessments identify potential inhibitors of L-GLS, including 6-diazo-5-oxo-L-norleucine and tannic acid, with higher binding affinities to bacterial L-GLS compared to human L-GLS. These findings hold promise for developing targeted anticancer therapies leveraging bacterial L-GLS inhibition. L-GLS inhibitors such as 6-diazo-5-oxo-L-norleucine and tannic acid work by binding to L-GLS enzymes, preventing them from functioning normally. This inhibition of L-GLS specifically targets their metabolic pathways, which are crucial for their survival and proliferation. By selectively inhibiting L-GLS, this compound can potentially disrupt cancer cells that rely on bacterial metabolism, opening up new possibilities for targeted anticancer therapies^74^. These findings align with previous studies highlighting the importance of residues like GLN514 and ASP511 in bacterial GLS for ligand stabilization^75^. The role of π-π stacking interactions, such as those observed with MET510, has been extensively discussed in earlier research on natural inhibitors^76^. The observed docking scores and interaction profiles support the hypothesis that tannic acid and DON may serve as selective inhibitors for bacterial GLS, with tannic acid demonstrating superior binding potential. Overall, these results provide a strong foundation for understanding the molecular basis of ligand specificity in bacterial versus human GLS. The observed selectivity of tannic acid for bacterial L-GLS over human L-GLS (0.99 kcal/mol binding energy difference) provides crucial evidence for its potential as a targeted anticancer therapy. This selectivity is attributed to the higher density of polar residues in the bacterial L-GLS binding pocket, facilitating stronger hydrogen bonding interactions, while the human L-GLS binding pocket exhibits less optimal spatial alignments. This differential binding profile suggests that tannic acid can preferentially target cancer cells utilizing bacterial L-GLS while minimizing adverse effects on normal cellular L-GLS activity.

It was known that glutaminase plays a crucial role in cancer cell metabolism by catalyzing the conversion of glutamine to glutamate, which fuels various biosynthetic pathways essential for tumor growth and survival^77^. Inhibition of glutaminase activity has emerged as a promising strategy for cancer therapy, particularly in tumors reliant on glutamine metabolism^78,79^. As described in the study, the docking analysis identified tannic acid as a potential inhibitor of L-GLS. The higher binding affinity of tannic acid to L-GLS suggests its ability to inhibit GLS activity could disrupt cancer cell metabolism and hinder tumor progression. Leveraging tannic acid’s inhibitory effects on L-GLS could offer a targeted approach for disrupting cancer cell metabolism while minimizing off-target effects on normal tissues^80^. The findings regarding potential inhibitors of L-GLS in the current study align with similar computational studies targeting other enzymes^81,82^ and highlight the effectiveness and reliability of in silico assessments in drug discovery and development.

It is vital to acknowledge the study’s limitations. Firstly, this study utilized a one-factor-at-a-time (OFAT) approach to optimize key parameters, including incubation time, temperature, pH, and carbon and nitrogen sources for L-GLS enzyme production. While this approach provided valuable insights, it does not account for potential interactions among variables. Future studies should employ advanced statistical experimental designs, such as response surface methodology (RSM) or factorial design, to systematically evaluate these interactions and optimize the process more efficiently. Incorporating such models would enable a more comprehensive understanding of synergistic effects between variables and ensure a more robust and reproducible optimization process. While this study successfully evaluated the docking scores and interactions of the ligands with the target proteins, ligand geometry optimization was not performed after obtaining the compounds. This step is crucial for ensuring the ligands are in their energetically favorable conformations, which can significantly enhance the reliability and accuracy of docking predictions. Before docking analysis, ligand geometry optimization will be incorporated in future studies using appropriate computational tools, such as density functional theory (DFT) or molecular mechanics. This addition will allow for a more precise evaluation of binding interactions and contribute to a deeper understanding of the ligand-protein dynamics.

## Conclusion

Our study comprehensively investigated the production, purification, and preliminary therapeutic evaluation of L-GLS derived from *Halomonas aquamarina* HBIM1. Key findings include the identification of WS6 as a highly efficient bacterial isolate, optimal production conditions utilizing fructose and ammonium sulfate as preferred carbon and nitrogen sources, and successful purification methods that enhanced catalytic efficiency and purity as confirmed by SDS-PAGE analysis. Kinetic analysis revealed optimal pH and thermal stability conditions for L-GLS activity, with favorable substrate affinity (Km = 0.198 mM⁻¹). In vitro cytotoxicity evaluation demonstrated selective antiproliferative effects on HepG2 liver cancer cells (IC50 = 33.98 µg/ml) compared to normal WI-38 cells (IC50 = 93.43 µg/ml), indicating a 2.75-fold selectivity index that warrants further investigation. However, these preliminary in vitro findings require comprehensive validation through preclinical studies to establish in vivo efficacy, safety profile, and pharmacological properties before considering therapeutic applications. Computational analysis identified tannic acid and 6-diazo-5-oxo-L-norleucine (DON) as potential selective inhibitors with higher binding affinities to bacterial L-GLS compared to human L-glutaminase (selectivity indices of 5.0 and 3.5, respectively), suggesting possibilities for combination therapy approaches that merit preclinical validation. While our findings provide proof-of-concept evidence for the anticancer potential of *Halomonas aquamarina* L-GLS, extensive preclinical studies, including in vivo efficacy evaluation, comprehensive toxicological assessment, pharmacokinetic profiling, and immunogenicity studies, are essential prerequisites before clinical translation.

## Data Availability

The datasets generated and/or analyzed during the current study are available in the GeneBank repository with accession number OR707005.1.
